# LBX2-AS1 Activates FSTL3 by Binding to Transcription Factor RARα to Foster Proliferation, Migration, and Invasion of Thyroid Cancer

**DOI:** 10.3389/fgene.2021.765033

**Published:** 2021-11-11

**Authors:** Jia Li, Jie Shen, Lan Qin, Dongyan Lu, Enci Ding

**Affiliations:** Department of Nuclear Medicine, Tianjin First Central Hospital, School of Medicine Nankai University, Tianjin, China

**Keywords:** thyroid cancer, lncRNA LBX2-AS1, RARα, Fstl3, malignant progression

## Abstract

**Background:** Thyroid cancer is a frequent endocrine tumor in women. It is of great significance to investigate the molecular mechanism of progression of thyroid cancer.

**Methods:** Gene expression data set and clinical data were downloaded from The Cancer Genome Atlas database for differential expression analysis. The triplet of downstream transcription factors (TFs) and modulatory genes of target lncRNA in thyroid cancer was predicted by the lncMAP database. mRNA and protein expression of lncRNA LBX2-AS1, RARα, and FSTL3 were detected by qRT-PCR and western blot. The localization of lncRNA LBX2-AS1 in cells was tested by Fluorescence in situ hybridization assay. The RNA immunoprecipitation assay was applied to verify the binding relationship between lncRNA LBX2-AS1 and FSTL3. ChIP and dual-luciferase assays were used to prove the binding relationship between RARα and FSTL3. Cell function experiments were used to test cell proliferation, migration and invasion in each treatment group. The role of lncRNA LBX2-AS1 in thyroid cancer progression was also confirmed in nude mice.

**Results:** Bioinformatics analysis indicated that lncRNA LBX2-AS1, RARα, FSTL3 were remarkably fostered in thyroid cancer tissue, and LBX2-AS1 was evidently correlated with clinical features. The LncMAP triplet prediction showed that LBX2-AS1 recruited TF RARα to modulate FSTL3. RIP assay confirmed that LBX2-AS1 was prominently enriched on RARα. ChIP and dual-luciferase report assays unveiled that RARα bound to the promoter region of FSTL3 and functioned as a TF. Cell function experiments uncovered that LBX2-AS1 boosted the progression of thyroid cancer. The rescue experiments showed that LBX2-AS1 recruited the TF RARα to hasten the transcription activity of FSTL3 and thus promoted the development of thyroid cancer.

**Conclusion:** The integrative results demonstrated that LBX2-AS1 activated FSTL3 by binding to TF RARα to hasten proliferation, migration and invasion of thyroid cancer.

## Introduction

LncRNAs are a class of key non-coding RNA involved in gene regulation. So far, lncRNA has been uncovered to be correlated with a variety of human diseases (especially cancer) (Guo et al., 2016; Zhang et al., 2019a). LncRNA acts as a co-activator to bind transcription factors (TFs) and boost their transcriptional activity to activate or suppress the transcription of specific targets (Caretti et al., 2006; Feng et al., 2006), which is the limelight of research currently. For instance, lncRNA TMPO-AS1 boosts the transcription activity of LCN2 by binding to the TF E2F6, thereby facilitating the development of ovarian cancer (Zhao et al., 2020). LBX2-AS1 is a key lncRNA involved in tumorigenesis. So far, many studies have uncovered that LBX2-AS1 plays a part in fostering the development of cancer. For instance, lncRNA LBX2-AS1 promotes human cancers like glioma (Chen et al., 2020), gastric cancer (Peng et al., 2020), esophageal squamous cell carcinoma (Zhang et al., 2019b), colorectal cancer (Li et al., 2021), and ovarian cancer (Cao et al., 2021). Many studies have dug the role of lncRNA LBX2-AS1 in cancers, but there are few studies on the effect of lncRNA LBX2-AS1 in thyroid cancer by recruiting TF to modulate downstream mRNA. As a result, studying the function of lncRNA-TF-mRNA regulation axis in thyroid cancer is of great significance for targeted drug treatment of thyroid cancer.

The molecular mechanism of LncRNA-TF-mRNA is often explored in cancers. For example, lncRNA TMPO-AS1 hastens the transcriptional activity of LCN2 by binding to the TF E2F6, thereby boosting the development of ovarian cancer (Zhao et al., 2020). Our study revealed that the lncRNA LBX2-AS1/RARα/FSTL3 axis may affect the progression of thyroid cancer through bioinformatics method. RARα, a nuclear receptor TF, plays a vital role during development processes and normal physiological functions (Khetchoumian et al., 2008; Zhu et al., 2010). RARα is differentially expressed in different tumor tissues, and it also interacts with its target genes to participate in tumor growth, metastasis, drug resistance and other processes (Altucci et al., 2007; Liu et al., 2012). For instance, RARα overexpression enhances the malignant transformation during mammary tumorigenesis (Doi et al., 2015). RARα up-regulating EGFR expression can lead to resistance to 5-FU in colon cancer (Gu et al., 2020). FSTL3 is a new type of cytokine that can regulate insulin sensitivity and counteract the signal transduction of activin or myostatin. Studies have proved that FSTL3 is involved in modulating tumor progression. For instance, FSTL3 overexpression accelerates progression of non-small cell lung cancer cells (Gao et al., 2020). FSTL3 is elevated and hastens tumor cell proliferation by antagonizing endogenous activin in invasive breast cancer (Razanajaona et al., 2007). Although some studies focused on the effects of RARα and FSTL3 on tumor development, much less has been understood on RARα/FSTL3 axis regulation on tumor.

Combining the previous research and bioinformatics research results, this study speculated that the lncRNA LBX2-AS1/RARα/FSTL3 axis can affect the development and progression of thyroid cancer. Subsequently, molecular experiments, cell experiments and animal model experiments confirmed that lncRNA LBX2-AS1 can recruit the TF RARα to foster the expression of FSTL3 to boost development of thyroid cancer. This finding can lay a foundation for the future development of targeted drugs for thyroid cancer.

## Materials and Methods

### Bioinformatics Approaches

The lncRNA-seq expression data set (normal: 58, tumor: 510) and the clinical data of thyroid cancer were acquired from The Cancer Genome Atlas (TCGA) database (https://www.cancer.gov/about-nci/organization/ccg/research/structural-genomics/tcga). The normal sample was applied as the control group, and differential analysis was carried out using R package “edgeR” (|logFC| > 1, FDR < 0.05). The lncATLAS database (https://lncatlas.crg.eu/) was employed to analyze the subcellular localization of the target lncRNA. Through the lncMAP database (http://bio-bigdata.hrbmu.edu.cn/LncMAP/index.jsp), the triplet of downstream TFs and regulatory genes of target lncRNA in thyroid cancer was predicted. Meanwhile, RNA-Protein Interaction Prediction was utilized to evaluate the possibility of interaction between lncRNA and TF. JASPAR database (http://jaspar.genereg.net/) was adopted to predict the binding sequences of target gene promoter region and TF (the first 2000 bp of target gene transcription start position was selected).

### Cell Cultivation

Normal human thyroid cell line HTori-3 (BNCC338687), human thyroid cancer cell lines TPC-1 (BNCC338689), KTC-1 (BNCC340144), and FTC-133 (BNCC337959) were bought from BeNa Culture Collection (Shanghai, China). The above cell lines were cultivated in Roswell Park Memorial Institute (RPMI)-1640 medium plus 10% fetal bovine serum (FBS) at routine culture conditions.

### Cell Transfection

si-LBX2-AS1, oe-LBX2-AS1, oe-FSTL3, si-RARα, sh-LBX2-AS1, and the corresponding negative controls were all provided by Ribobio (China). In accordance with the operating instructions, si-LBX2-AS1, oe-LBX2-AS1, oe-FSTL3, si-RARα, sh-LBX2-AS1, and the corresponding negative controls were transfected into TPC-1 and KTC-1 using Lipofectamine 2000 kit (Themo Fisher, United States).

### Fluorescence *in Situ* Hybridization

KTC-1 cells were seeded in a confocal dish for 24 h. Then the cells were fixed, pre-hybridized, and hybridized with LncRNA LBX2-AS1 oligodeoxynucleotide probe (GenePharma, Shanghai, China) in hybridization buffer overnight. The fluorescent FISH kit (GenePharma, China) was applied to detect the signal of the probe, then DAPI was adopted to stain the nucleus. Finally, the image was checked under a confocal microscope.

### qRT-PCR

TRIzol reagent (Life Technologies, United States) was utilized to extract total RNA from the cells, and then RNA concentration was measured using NanoDrop 2000 system (Thermo Fisher Scientific, Inc., United States). In line with the kit instructions, total RNA was reversely transcribed by PrimeScript RT Master Mix (Takara, P.R., Japan). The miScript SYBR Green PCR Kit (Qiagen, Germany) was applied to perform qRT-PCR on the Bio-Rad CFX96 real-time PCR detection system (Bio-Rad Laboratories, Hercules, United States) to test the expression levels of lncRNA LBX2-AS1, RARα, and FSTL3. β-actin served as a standardized endogenous control. The 2^−ΔΔCt^ value was conducted to compare the relative expression levels of lncRNA LBX2-AS1, RARα, and FSTL3. The primer sequences are listed in Table1.

**TABLE 1:** Primer sequences for qPCR.

Gene	Forward primer (5′-3′)	Reverse primer (5′-3′)
LBX2-AS1	AGTTTGTCCCAGGTT TGGCA	CATGCCAGGGTCCTT GTTCT
RARα	AGC​ACC​AGC​TTC​CAG​TTA​GTG​G	CAA​AGC​AAG​GCT​TGT​AGA​TGC​GG
FSTL3	ACA​TTG​ACA​CCG​CCT​GGT​CCA​A	ACT​CCA​CGC​CGT​CGC​ACG​AAT
β-actin	TCC​GGC​ACT​ACC​GAG​TTA​TC	GAT​CCG​GTG​TAG​CAG​ATC​GC

### CCK-8

Cell Counting Kit-8 (MedChemExpress, United States) was utilized to examine cell viability. The transfected cells were planted into 96-well plates, and 10 μl of CCK-8 detection reagent was supplemented after the cells were cultured for 1, 2, 3, and 4 days. The cells were then incubated for 2 h, and microplate reader was applied to measure the absorbance at 450 nm. Three biological experiments were repeated in each group.

### Scratch Healing Assay

When the cell confluence reached 80%, a 200 μl pipette tip was utilized to gently scratch a line on the culture dish. Phosphate buffer saline (PBS) was used to wash the culture dish twice briefly to remove cell debris, and then fresh serum-free medium was added to continue to culture the cells for 48 h. The scratch images at 0 and 48 h and the width of the scratches were photographed using a microscope to detect the migration of thyroid cancer cells. Relative wound sizes are calculated by normalizing the line widths by the width of the control group at 0 h.

### Transwell

Transwell assay was conducted to test the migration and invasion of thyroid cancer cells. Firstly, cells were seeded in the upper chamber pre-coated with Matrigel in a Transwell experimental device (Corning, United States) with an 8 μm pore size. Then, RPMI-1640 medium with 10% FBS was supplemented to the lower chamber. After the cells were cultured at 37°C for 1 day, a cotton swab was used to wipe the non-invading cells on the upper surface of the membrane. The migrating cells were fixed with 4% paraformaldehyde, and then stained with crystal violet for 30 min. Finally, five fields were randomly chosen under an optical microscope to take pictures and count the number of cells.

### Western Blot

The cells were lysed with radioimmunoprecipitation assay lysis buffer (Thermo Fisher, United States). Then the total proteins were extracted and BCA kit (Beyotime, China) was applied to test the protein concentration. Separated on sodium dodecyl sulfate-polyacrylamide gel electrophoresis (50 μg/lane), the protein samples were transferred to polyvinylidene fluoride membrane. At room temperature, 5% skimmed milk was employed to seal the membrane for 2 h. Then, the membrane was cultivated with primary antibodies overnight at 4°C. Afterwards, the membrane was incubated with secondary antibody for 2 h at room temperature. Finally, the hypersensitive chemiluminescence kit (Thermo Fisher Scientific) was utilized to visualize the protein on the membrane. The primary antibodies utilized in this study were mainly anti-β-actin, anti-RARα, anti-FSTL3, anti-N-cadherin, anti-Vimentin, anti-MMP2, and anti-MMP9 (Invitogen, United States). The secondary antibody used was horseradish peroxidase-(HRP-) labeled goat anti-rabbit IgG (Invitogen, United States).

### RNA Immunoprecipitation

Binding of LBX2-AS1 to RARα was explored using RIP kit (millipore, United States). Pre-cooled PBS was applied to wash the KTC-1 cells and then the supernatant was discarded. An appropriate amount of lysis buffer (P0013B, Beyotime) was employed to lyse the cells, and then cells were centrifuged at 14,000 rpm and 4°C for 10 min. A portion of the cell extract was taken out as the input group, and another portion was incubated with the magnetic bead-antibody complex for co-precipitation. The specific experimental operation method was as follows: 50 μl magnetic beads was taken from each co-precipitation reaction system, washed and resuspended in 100 μl RIP Wash Buffer. 5 μg antibody was added for binding. After being washed, the magnetic bead-antibody complex was resuspended in 900 μl RIP Wash Buffer, and 100 μl cell extract was added for incubation at 4°C overnight. The sample was placed on a magnetic stand to collect the magnetic bead-protein complex. After the sample was digested with proteinase K, RNA was extracted for subsequent qRT-PCR detection. anti-RARα (Invitogen, United States) was used in RIP and mixed at room temperature for 30 min. IgG (Invitogen, United States) was served as a negative control.

### Chromatin Immunoprecipitation Assay

Firstly, KTC-1 cells were transfected with pCMV-RARα. After 24 h of transfection, ChIP assay was carried out using IP-grade anti-RARα antibody (Invitrogen, United States) and the corresponding simple ChIP enzymatic chromatin IP kit (CST, United States). qPCR was applied to detect purified DNA. The detection primers used are shown in Table 2.

**TABLE 2:** Primer sets for ChIP assay.

Primer sets	Sequence (5′-3′)
RARα upstream	
Forward	AGC​ACC​AGC​TTC​CAG​TTA​GTG​G
Reverse	CAA​AGC​AAG​GCT​TGT​AGA​TGC​GG

### Dual-Luciferase Assay

The pmirGLO-FSTL3-promoter-wild type (WT) and pmirGLO-FSTL3-promoter-mutant (MUT) luciferase reporter vectors (Promega, United States) were transfected. Then, the thyroid cancer cell line KTC-1 was seeded in 96-well plates. FSTL-WT and FSTL-MUT, si-NC and si-LBX2-AS1, oe-NC, and oe-RARα plasmids were co-transfected with the cells. After 48 h of incubation, the luciferase intensity was measured using the dual-luciferase reporter system (Promega, United States).

### Immunohistochemistry Assay and Hematoxylin-Eosin Staining

The HE staining was performed according to the following steps: the tumor was fixed in 4% paraformaldehyde solution (Aladdin, China) for 48 h at room temperature, dehydrated, and embedded in paraffin. A microtome was applied to slice the tumor with a thickness of 4 μm, and then the slices were stained with HE reagent after deparaffinization.

In the IHC experiment, the paraffin slices in the previous steps were first deparaffinized, and then 3% hydrogen peroxide was added for incubation to eliminate peroxidase activity. The slices were arrested with 5% goat serum, and then were incubated with Ki67 antibody (Abcam, Cambridge, United Kingdom) at 4°C overnight. The next day, the slices were incubated with the secondary antibody goat anti-mouse IgG H and L (Abcam, Cambridge, United Kingdom) at 37°C for 1 h. After incubation, the slices were washed with washing buffer (Beyotime, China) at room temperature, and then developed using DAB kit (Solarbio, DA1010, China). Finally, the slices were observed and photographed.

### Tumor Xenograft

To perform tumor xenograft assay, KTC-1 cells were transfected with sh-NC and sh-LBX2-AS1 stably. Subsequently, 10 nude mice (4–5 weeks) were randomly divided into groups (*n* = 5) and then injected subcutaneously with the harvested cells. The tumor volume of nude mice was measured every 5 days, and calculated according to the formula (x (Zhang et al., 2019a) × y)/2 (x = width; y = length). After 40 days, the mice were sacrificed. The tumor weight was measured and further analyzed.

### Statistical Analysis

All data were dealt with SPSS 22.0 statistical software. Measurement data were expressed as mean ± standard deviation. The comparison between the two groups used was *t-*test, and the comparison among multiple groups adopted was one-way analysis of variance. *p* < 0.05 illustrated that the difference was statistically significant.

## Results

### LncRNA LBX2-AS1 is Evidently Up-Regulated in Thyroid Cancer Tissue and Cells

**FIGURE 1:**
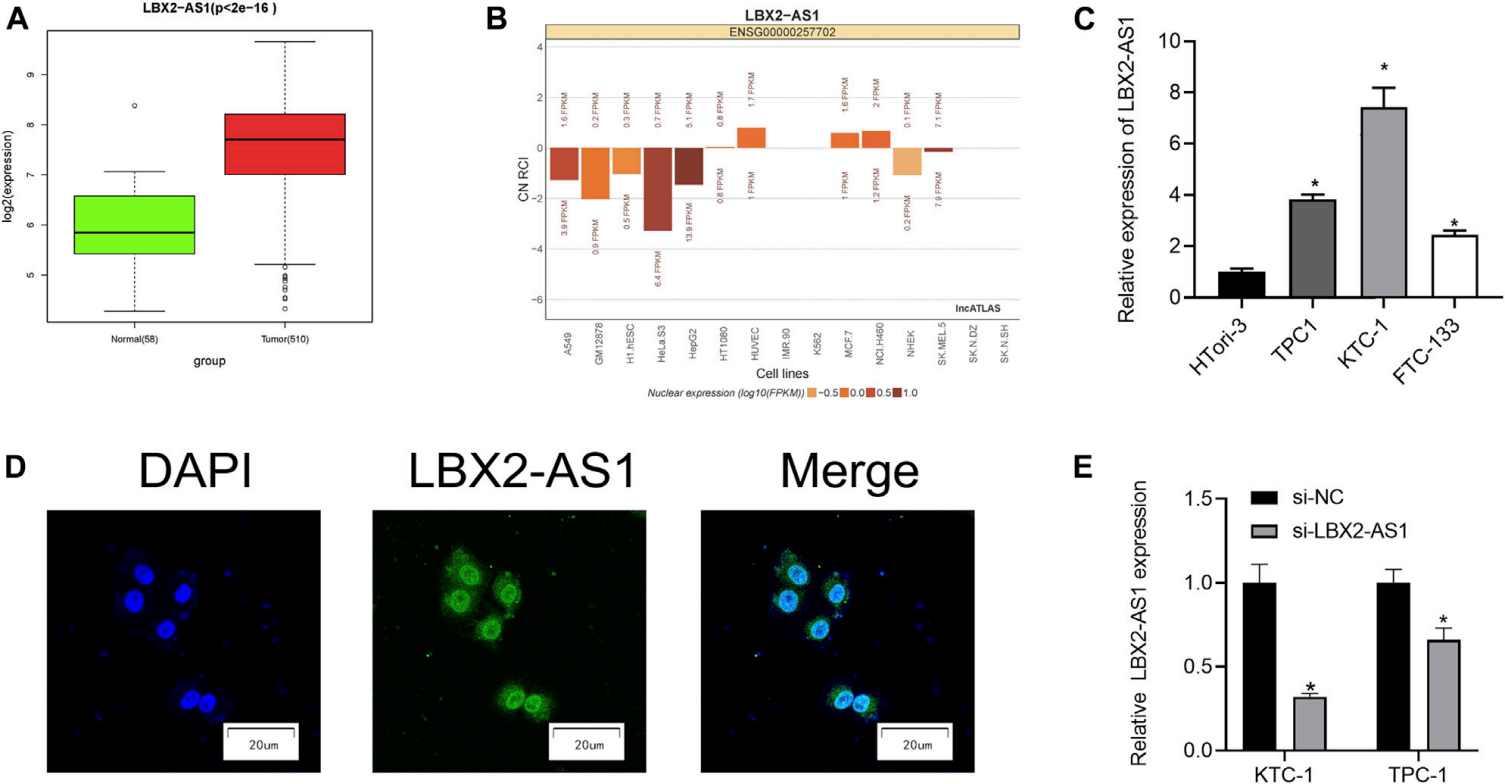
LncRNA LBX2-AS1 is evidently activated in thyroid cancer tissue and cells. **(A)** Expression of lncRNA LBX2-AS1 in normal and tumor tissue samples; **(B)** The subcellular localization results of lncRNA LBX2-AS1 in 15 cell lines, based on the CN RCI value; **(C)** LncRNA LBX2-AS1 expression in thyroid cancer cell lines TPC-1, KTC-1, FTC-133, and human normal thyroid cell line HTori-3; **(D)** Localization of lncRNA LBX2-AS1 in thyroid cancer cells, scale bar = 20 μm; **(E)** Transfection efficiency of si-LBX2-AS1 in KTC-1 and TPC1 cell lines; *p < 0.05.

As revealed by informatics results, compared with normal thyroid tissue, LBX2-AS1 was prominently boosted in thyroid cancer tissue (Figure 1A), and LBX2-AS1 was correlated with clinical features (T, N, Stage) (Table 3). The subcellular localization results indicated that LBX2-AS1 was expressed in both cytoplasm and nucleus (Figure 1B). In combination with the results of previous studies (Tang et al., 2019; Li et al., 2021), we noted that LBX2-AS1 boosted malignant progression of thyroid cancer. To verify its expression level in thyroid cancer, we performed qRT-PCR and found that LBX2-AS1 was markedly up-regulated in thyroid cancer cell lines, and highly expressed in TPC1 and KTC-1 cell lines (Figure 1C). Subsequently, it was proved that lncRNA LBX2-AS1 was expressed both in nucleus and cytoplasm by FISH (Figure 1D). To further identify the function of LBX2-AS1 in thyroid cancer, si-LBX2-AS1 was transfected into thyroid cancer cells, and then qRT-PCR verified the transfection efficiency. The experimental results revealed that cells transfected with si-LBX2-AS1 showed remarkably descended expression level of LBX2-AS1 and good transfection efficiency (Figure 1E), indicating that the cells could be utilized for subsequent functional verification experiments.

**TABLE 3:** The relationship between LBX2-AS1 expression and the clinicopathological features of patients with thyroid cancer.

	Low expression LBX2-AS1 (N = 250)	High expression LBX2-AS1 (N = 249)	*p*-value
**Age (years)**			
age <65	209 (83.6%)	214 (85.9%)	0.546
age≥65	41 (16.4%)	35 (14.1%)	
**Event**			
Yes	10 (4.0%)	6 (2.4%)	0.451
No	240 (96.0%)	243 (97.6%)	
**T**			
T1	80 (32.0%)	60 (24.1%)	0.00229
T2	92 (36.8%)	72 (28.9%)	
T3	72 (28.8%)	98 (39.4%)	
T4	5 (2.0%)	18 (7.2%)	
TX	1 (0.4%)	1 (0.4%)	
**N**			
N0	127 (50.8%)	101 (40.6%)	<0.001
N1	91 (36.4%)	131 (52.6%)	
NX	32 (12.8%)	17 (6.8%)	
**M**			
M0	130 (52.0%)	151 (60.6%)	0.0621
M1	3 (1.2%)	6 (2.4%)	
MX	117 (46.8%)	92 (36.9%)	
**stage**			
Stage I	151 (60.4%)	130 (52.2%)	<0.001
Stage II	36 (14.4%)	16 (6.4%)	
Stage III	47 (18.8%)	64 (25.7%)	
Stage IV	16 (6.4%)	39 (15.7%)	

* Means death event.

### LncRNA LBX2-AS1 Fosters Progression of Thyroid Cancer Cells

**FIGURE 2:**
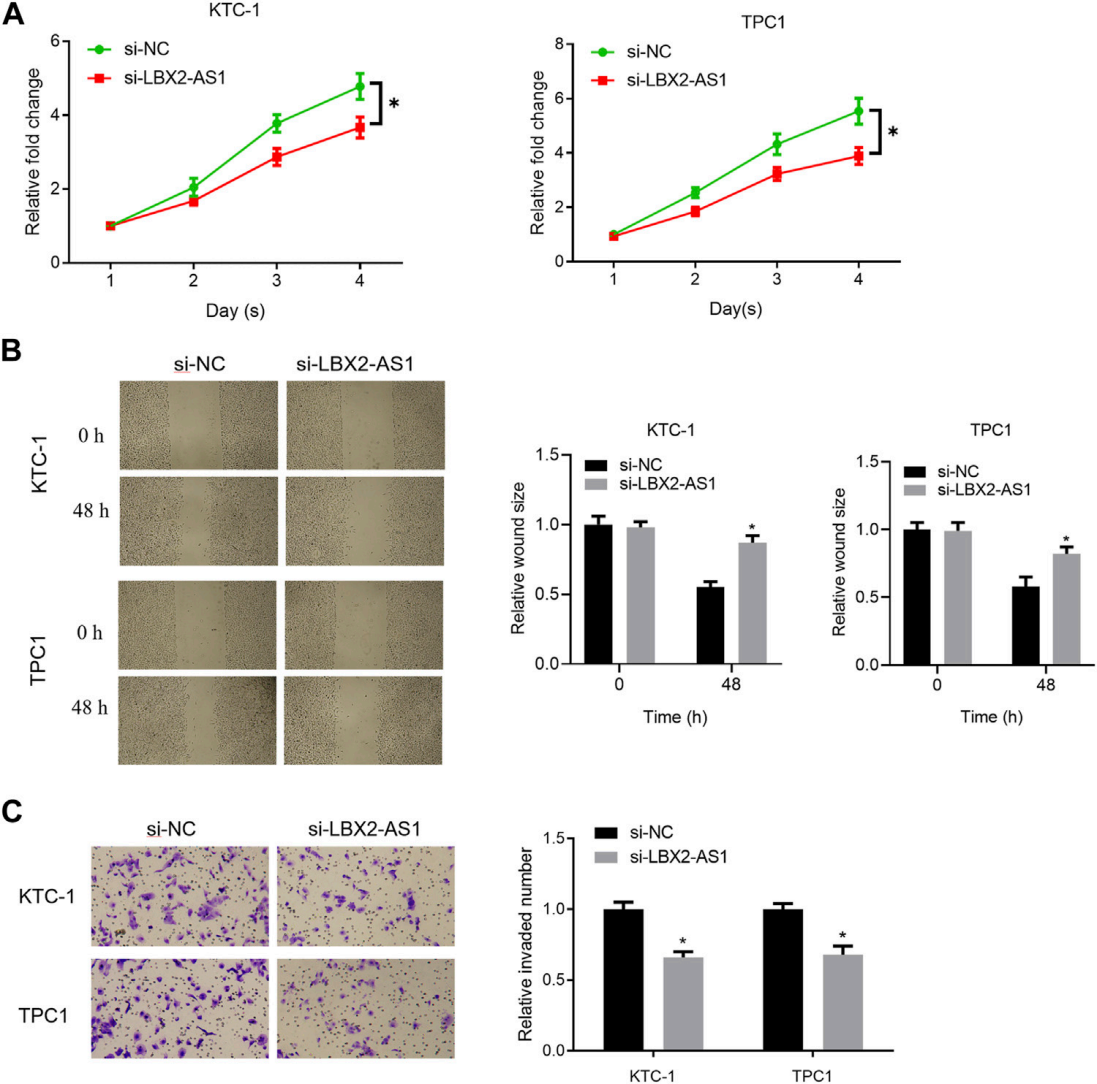
LncRNA LBX2-AS1 fosters progression of thyroid cancer cells. **(A)** CCK-8 detected viability of KTC-1 and TPC1 cells; **(B)** Scratch healing tested invasion of KTC-1 and TPC1 cells; **(C)** Transwell detected invasion of KTC-1 and TPC1 cells; *p < 0.05.

In the previous study, it was found that level of LBX2-AS1 in thyroid cancer tissue and cells was prominently hastened. Based on the literature, we speculated that LBX2-AS1 may promote thyroid cancer (Yang et al., 2021). To verify the above conjecture, we further tested proliferation, migration and invasion of thyroid cancer cells. The results showed that silencing LBX2-AS1 markedly reduced viability of KTC-1 and TPC1 cells (Figure 2A). The results of scratch healing and Transwell assays uncovered that silencing LBX2-AS1 in KTC-1 and TPC1 cells evidently suppressed migration and invasion of thyroid cancer cells (Figures 2B,C). Concludingly, LBX2-AS1 promoted malignancy of thyroid cancer process.

### LncRNA LBX2-AS1 Modulates Progression of Thyroid Cancer Cells via Regulating FSTL3

**FIGURE 3:**
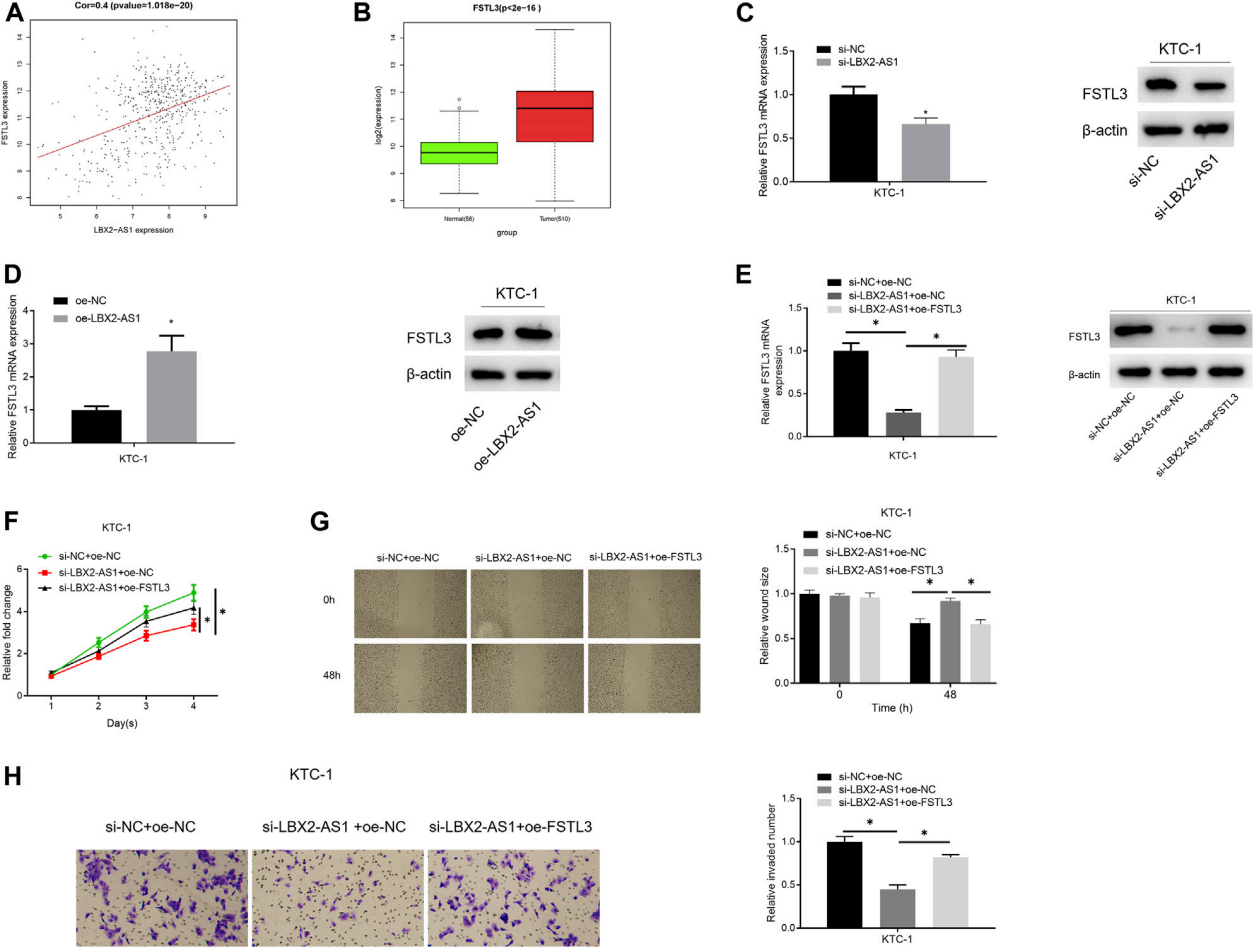
lncRNA LBX2-AS1 modulates progression of thyroid cancer cells via regulating FSTL3. **(A)** Pearson correlation analysis of LBX2-AS1 and FSTL3; **(B)** FSTL3 expression in normal tissue and tumor tissue; **(C–E)** qRT-PCR and western blot detected the expression of FSTL3 mRNA and protein in different transfection groups; **(F)** CCK-8 method detected viability of KTC-1 cells in different transfection groups; **(G)** Scratch healing detected migration of KTC-1 cells in different transfection groups; **(H)** Transwell detected invasion of KTC-1 cells in different transfection groups; *p < 0.05.

The results of bioinformatics indicated a significantly positive correlation between FSTL3 and LBX2-AS1 (Figure 3A), and FSTL3 was evidently highly expressed in tumor tissue (Figure 3B). Combining a previous study (Gao et al., 2020), it was believed that lncRNA LBX2-AS1 regulates the progression of thyroid cancer through FSTL3. To verify it, oe-FSTL3, oe-LBX2-AS1, and si-LBX2-AS1 plasmids were used to transfect KTC-1 cells, respectively. As expressed in the experimental results, FSTL mRNA and protein expression levels were prominently reduced after silencing LBX2-AS1 (Figure 3C), while LBX2-AS1 overexpression significantly increased FSTL mRNA and protein expression levels (Figure 3D). In addition, overexpressing FSTL and silencing LBX2-AS1 meanwhile could restore FSTL3 mRNA and protein expression levels (Figure 3E), as well as the proliferation, migration and invasion of KTC-1 cells (Figures 3F–H). These results further confirmed that LBX2-AS1 modulated progression of thyroid cancer cells through FSTL3.

### LncRNA LBX2-AS1 can Recruit RARα

**FIGURE 4:**
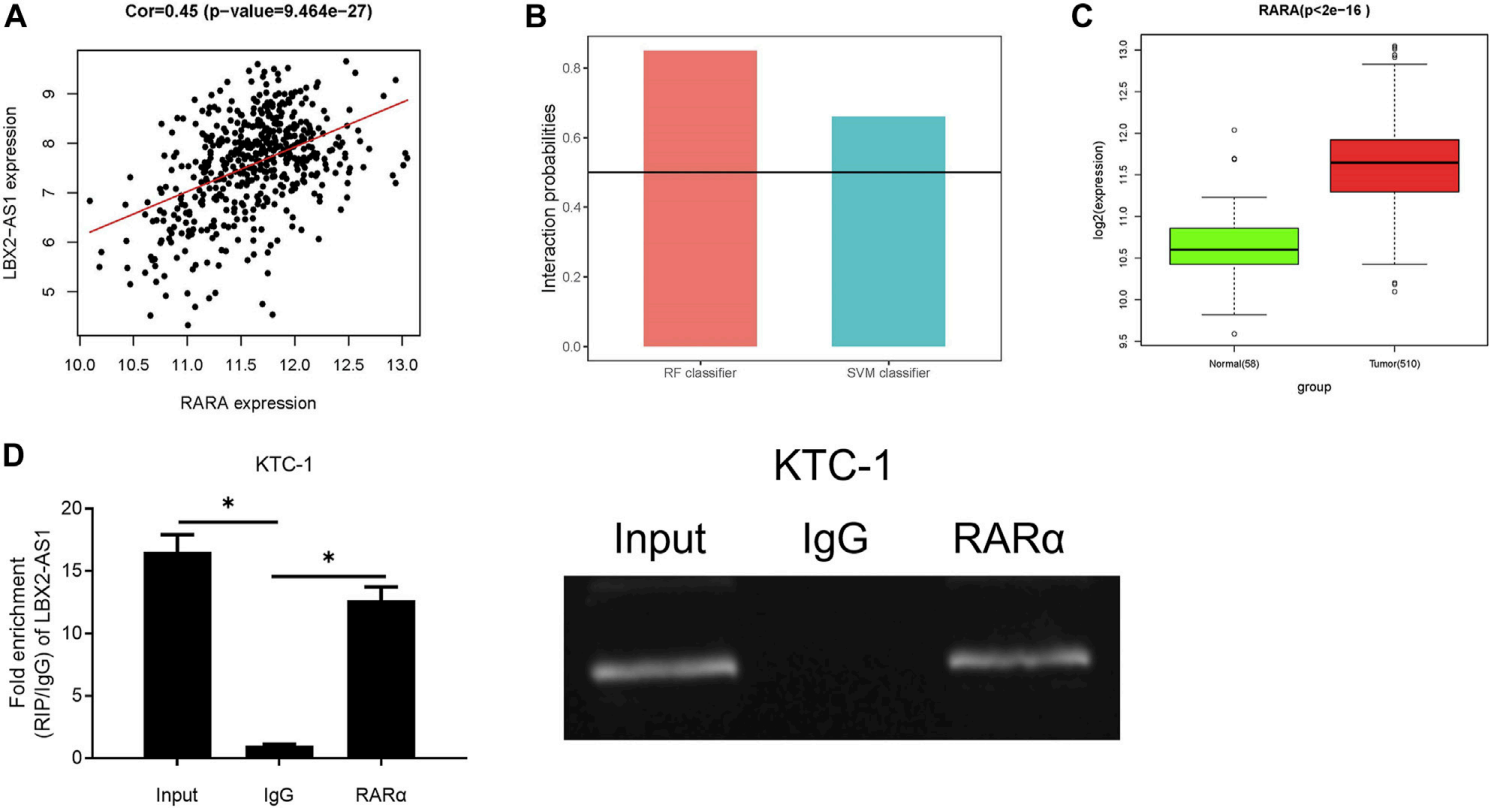
LncRNA LBX2-AS1 can recruit RARα. **(A)** Heat map of Pearson correlation analysis between RARα and LBX2-AS1; **(B)** LBX2-AS1 and RARα protein interaction score predicted by RPISeq database. It is predicted that LBX2-AS1 binds to RAR protein in the case of both the SVM and RF scores are higher than 0.5 simultaneously; **(C)** Box plot of RARα expression in normal and tumor groups; **(D)** RIP assay on KTC-1 cells using RARα and IgG antibodies; *p < 0.05.

Many lncRNAs has been reported to participate in molecular regulatory pathways by interacting with DNA or RNA-binding proteins. In an effort to explore molecular mechanism of regulatory effect of LBX2-AS1 on FSTL3, we analyzed correlation between RARα and LBX2-AS1 expression. Result revealed that RARα was markedly positively correlated with LBX2-AS1 (Figure 4A). At the same time, the RPISeq database score indicated that LBX2-AS1 interacted with RARα with high reliability (Figure 4B), and RARα was prominently boosted in thyroid cancer tissue (Figure 4C). Therefore, we speculated that RARα, as a TF, may be recruited to the FSTL3 promoter by LBX2-AS1. To confirm it, we conducted RIP assay, and the results suggested that LBX2-AS1 was prominently bound to RARα (Figure 4D).

### LBX2-AS1 Modulates FSTL3 by Recruiting RARα to Hasten Progression of Thyroid Cancer

**FIGURE 5:**
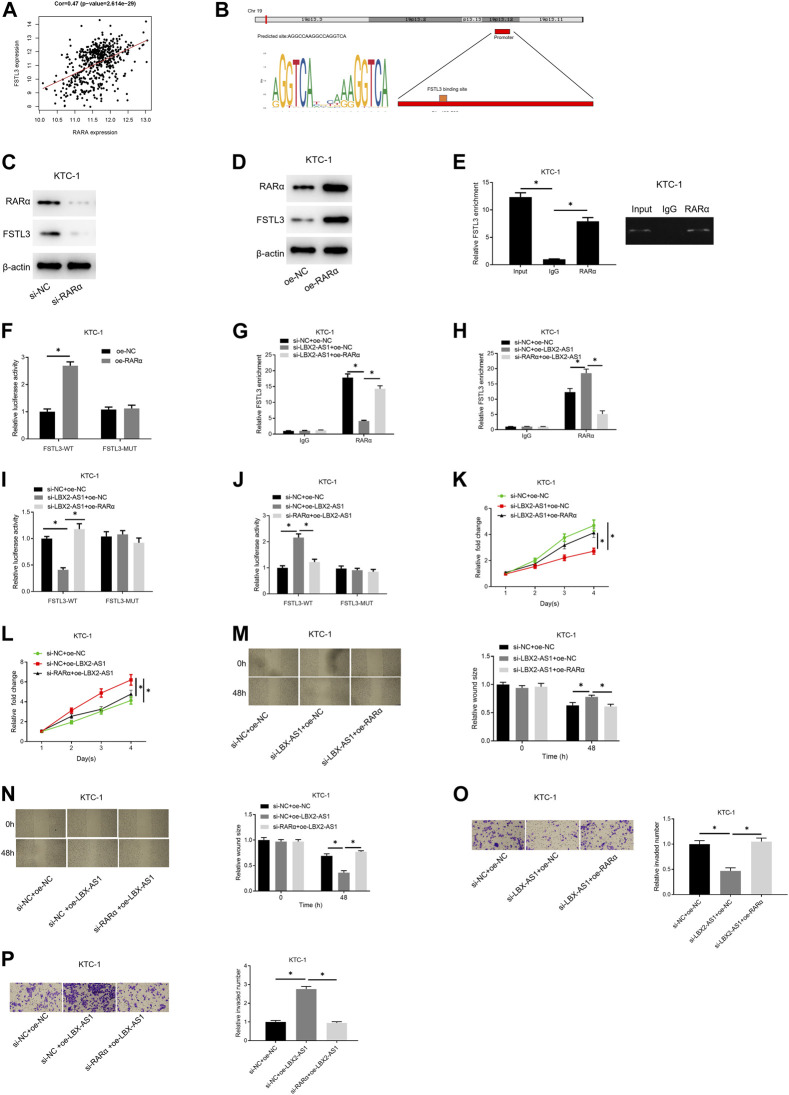
LBX2-AS1 modulates the expression of FSTL3 by recruiting RARα to hasten the progression of thyroid cancer. **(A)** Pearson correlation analysis between RARα and FSTL3; **(B)** The binding relationship between the FSTL3 promoter region and RARα was predicted by the JASPAR database; **(C,D)** Western blot detected FSTL3 protein expression in KTC-1 cells in different transfection groups; **(E)** RARα and IgG antibodies were used to perform RIP assay on KTC-1 cells; **(F)** Dual-luciferase assay detected the luciferase activity of KTC-1 cells in different transfection groups; **(G,H)** RARα and IgG antibodies were used to perform ChIP assay on KTC-1 cells; **(I,J)** Dual-luciferase assay detected the luciferase activity of KTC-1 cells in different transfection groups; **(K,L)** CCK-8 detected the cell viability of KTC-1 cells in different transfection groups; **(M,N)** Scratch healing detected the migration of KTC-1 cells in different transfection groups; **(O,P)** Transwell detected the invasion of KTC-1 cells in different transfection groups; *p < 0.05.

Correlation analysis confirmed an evident positive correlation between FSTL3 and RARα (Figure 5A). The JASPAR database was applied to examine the binding site of RARα and the TSS region of the FSTL3 promoter, finding that RARα had a binding site on the promoter (Figure 5B). To further study the regulation of RARα on the expression of FSTL3, we employed si-RARα and oe-RARα to treat KTC-1 cells, respectively. It was unveiled that si-RARα could reduce the expression of FSTL3 protein (Figure 5C) while oe-RARα increased the protein expression (Figure 5D). Subsequently, the binding relationship between RARα and the FSTL3 promoter was validated by ChIP assay. It was found that RARα could bind to the FSTL3 gene promoter (Figure 5E). To further confirm the binding relationship is functional, dual-luciferase assay was performed on KTC-1 cells. Overexpressing RARα increased the activity of WT FSTL3 luciferase, but had no effect on the activity of MUT FSTL3 luciferase (Figure 5F). Besides, silencing LBX2-AS1 reduced the enrichment of FSTL3 on RARα, but silencing LBX2-AS1 and overexpressing RARα meanwhile restored the enrichment (Figure 5G). On the contrary, overexpressing LBX2-AS1 enhanced the binding between RARα and FSTL3 promoter, but overexpressing LBX2-AS1 and silencing RARα meanwhile had an opposite effect (Figure 5H). In addition, the dual-luciferase assay further proved that silencing LBX2-AS1 reduced the WT FSTL3 luciferase activity, but silencing LBX2-AS1 and overexpressing RARα at the same time could restore the luciferase activity (Figure 5I). Overexpression of LBX2-AS1 enhanced WT FSTL3 luciferase activity, but overexpressing LBX2-AS1 while silencing RARα had an opposite effect (Figure 5J). We further tested the abilities of cell proliferation, migration, and invasion. As the experimental results indicated, silencing LBX2-AS1 markedly reduced the cell viability, migration, and invasion of KTC-1 cells, but when silencing LBX2-AS1 and overexpressing RARα at the same time, the inhibitory effect was offset (Figures 5K,M.,O). Moreover, overexpression of LBX2-AS1 prominently promoted cell viability, migration and invasion of KTC-1 cells, but when RARα was silenced and LBX2-AS1 was overexpressed meanwhile, the promotion effect was reversed (Figures 5L,N,P). These findings demonstrated that LBX2-AS1 modulated the expression of FSTL3 by recruiting the RARα to hasten the progression of thyroid cancer.

### The Impact of LBX2-AS1 on Tumors Is Verified in Nude Mice

**FIGURE 6:**
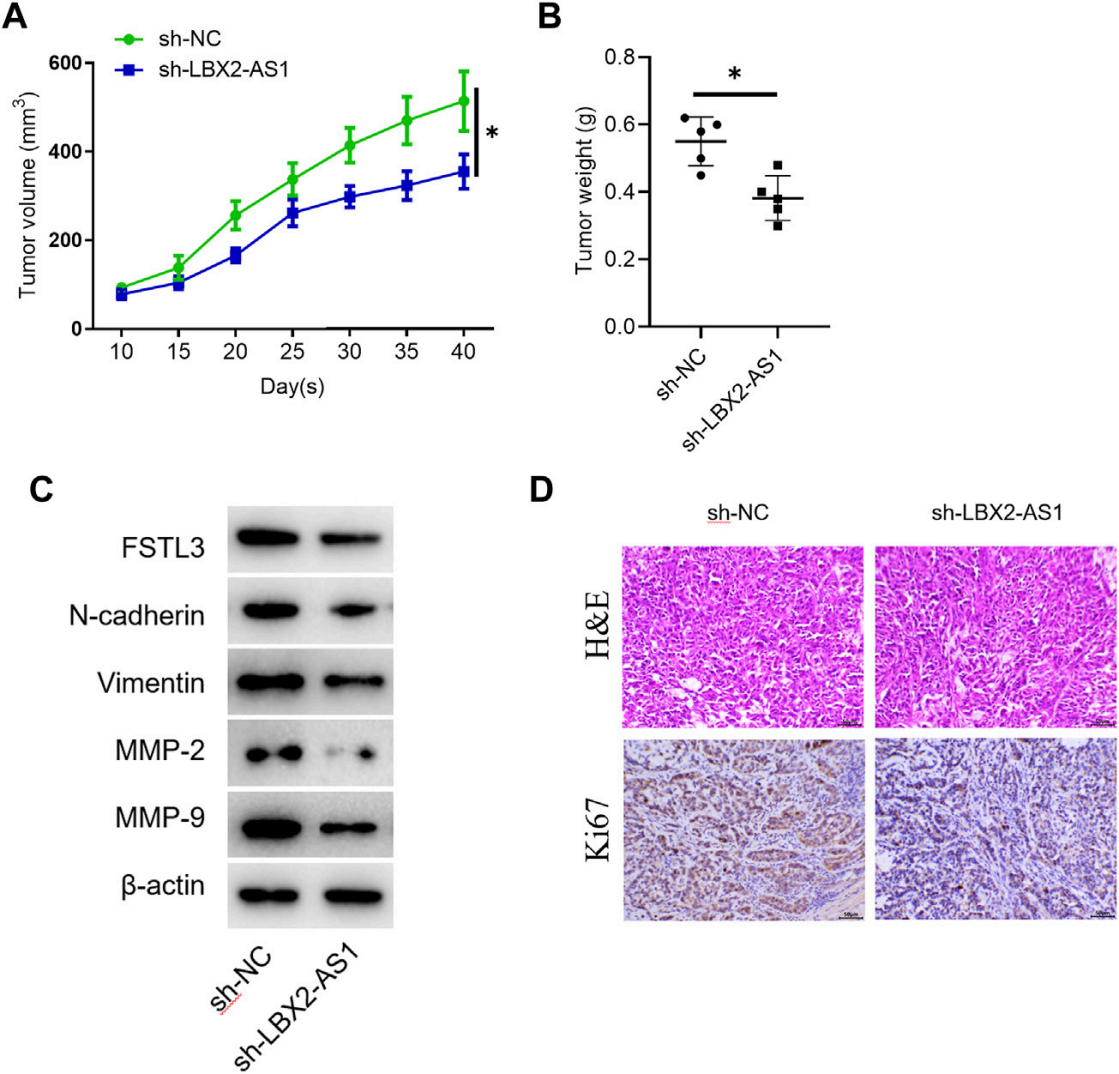
The effect of LBX2-AS1 on tumors is verified in nude mice. **(A)** Tumor volume in different transfection groups; **(B)** Tumor weight in different transfection groups; **(C)** Western blot detected protein expression of FSTL1 and invasion and migration related factors (N-cadherin, Vimentin, MMP-2, MMP-9) in nude mice; **(D)** IHC detected Ki67 expression in mice; *p < 0.05.

The tumor volume and weight of mice inoculated with sh-LBX2-AS1 transfected cells were reduced (Figures 6A,B). Western blot showed that the protein expression levels of FSTL3, Vimentin, N-cadherin, MMP2 and MMP-9 in the mice tumor tissue in the sh-LBX2-AS1 experimental group were decreased (Figure 6C), indicating that FSTL3 expression was increased at protein level and EMT was promoted. In addition, it was detected by IHC that silencing LBX2-AS1 decreased the expression of Ki67, indicating that the proliferation of thyroid cancer cells inoculated subcutaneously in mice was inhibited when LBX2-AS1 was silenced (Figure 6D). These findings suggested that LBX2-AS1 can be utilized as a potential therapeutic target of thyroid cancer.

## Discussion

In recent years, it has been discovered that lncRNAs can be employed as new and potential biomarkers for the prognosis and treatment of human cancers. The role and modulatory mechanism of lncRNAs in human cancers have become a research hotspot. Studies have confirmed a closely correlation between abnormal expression of lncRNA LBX2-AS1 and progression of tumors. For instance, LBX2-AS1 promotes E2F2 expression through sponge adsorption of miR-455-5p and miR-491-5p, thereby fostering progression of ovarian cancer (Cao et al., 2021). Overexpression of lncRNA LBX2-AS1 boosts the proliferation of colorectal cancer (Li et al., 2021). Herein, we revealed and verified that LBX2-AS1 was prominently highly expressed in thyroid cancer through bioinformatics analysis and cell biological experiments. Moreover, the results of cell function experiments showed that LBX2-AS1 could hastened progression of thyroid cancer cells, which is consistent with previous studies.

FSTL3 plays a part in cancers as a new type of cytokine. A study found that FSTL3 overexpression enhances proliferation and migration of non-small cell lung cancer cells (Gao et al., 2020). FSTL3 is facilitated in invasive breast cancer and can boost tumor cell proliferation by antagonizing endogenous activators (Razanajaona et al., 2007). Here, FSTL3 was prominently highly expressed in thyroid cancer tissue through bioinformatics analysis. Subsequently, cell experiments and molecular experiments displayed that after LBX2-AS1 was silenced, FSTL mRNA and protein expression levels were markedly reduced, and the proliferation, migration and invasion of KTC-1 cells were also suppressed. In addition, it was further confirmed by rescue experiments that LBX2-AS1 regulated progression of thyroid cancer cells through FSTL3.

Bioinformatics analysis suggested that there was a new TF RARα in the downstream of LBX2-AS1. At present, there are still few reports on RARα as a cancer factor. A study displayed that the overexpression of RARα enhances the malignant transformation during mammary tumorigenesis (Doi et al., 2015). In addition, another study indicated that RARα promotes the interaction of estrogen receptor coactivators and participates in estrogen-induced gene transcription in breast cancer cells (Ross-Innes et al., 2010). Bioinformatics analysis found that RARα was evidently elevated in thyroid cancer tissue and it was positively correlated with LBX2-AS1. Subsequently, RIP assay validated the binding relationship between LBX2-AS1 and RARα.

As a coactivator, lncRNA binds to TFs and enhances the transcriptional activity to activate or suppress the transcription of specific targets (Caretti et al., 2006; Feng et al., 2006), which is one of the regulatory mechanisms that have been studied more currently. For instance, LncRNA-BX111 recruits the TF YB1 to regulate the transcription of ZEB1 to foster the metastasis and progression of pancreatic cancer (Deng et al., 2018). It was demonstrated lncRNA TMPO-AS1 potentially fosters LCN2 transcriptional activity by binding to TF E2F6, and thus, stimulates the progression of ovarian cancer (Zhao et al., 2020). In this study, combined with the bioinformatics analysis and research of predecessors, it was speculated that LBX2-AS1 modulated the expression of FSTL3 by recruiting RARα to hasten the progression of thyroid cancer. ChIP assay and dual-luciferase report assay verified the binding relationship between RARα and FSTL3 gene promoter. Through cell and molecular experiments, it was revealed that silencing LBX2-AS1 reduced the enrichment of RARα on FSTL3, but silencing LBX2-AS1 and overexpressing RARα simultaneously had the opposite effect. In addition, the dual-luciferase assay further proved that silencing LBX2-AS1 could reduce the WT FSTL3 luciferase activity, but silencing LBX2-AS1 and overexpressing RARα meanwhile had the opposite effect. We further tested the cell proliferation, migration and invasion. As uncovered in the experimental results, silencing LBX2-AS1 prominently reduced the cell viability, migration and invasion of KTC-1 cells, but when silencing LBX2-AS1 and overexpressing RARα meanwhile, the inhibitory effect was restored. *In vivo* experiments in nude mice confirmed our conjecture, indicating that LBX2-AS1 modulated the expression of FSTL3 by recruiting RARα to accelerate the progression of thyroid cancer.

Collectively, this study uncovered the LBX2-AS1/RARα/FSTL3 modulatory axis in thyroid cancer. Subsequently, through cell experiments, molecular experiments, and *in vivo* experiments, it was unveiled that LBX2-AS1 boosts the transcriptional activity of FSTL3 by recruiting the binding of RARα, thereby hastening progression of thyroid cancer cells. This study discovered the molecular mechanism of LBX2-AS1 function in promoting cancer in thyroid cancer, providing a theoretical basis for the progression of targeted drugs for thyroid cancer.

## Data Availability

The original contributions presented in the study are included in the article/Supplementary Material, further inquiries can be directed to the corresponding author.
